# Comparison of follicular development, timing of ovulation and serum progesterone, estradiol and luteinizing hormone concentrations in dairy heifers treated with 4‐ or 5‐day CoSynch + CIDR protocols

**DOI:** 10.1002/vms3.171

**Published:** 2019-04-22

**Authors:** Heidi Fishman‐Holland, Agne Stoskute, Maria S. Ferrer, Deana Veal, Joao H. J. Bittar, Emmanuel Rollin, Jeferson Lourenço, Roberto A. Palomares

**Affiliations:** ^1^ Department of Large Animal Medicine College of Veterinary Medicine The University of Georgia Athens Georgia USA; ^2^ Department of Population Health College of Veterinary Medicine The University of Georgia Athens Georgia USA; ^3^ Department of Animal and Dairy Science College of Agriculture The University of Georgia Athens Georgia USA

**Keywords:** Co‐Synch, CIDR, Dairy heifer, Luteinizing hormone, Estradiol, Ovulation

## Abstract

The use of 4‐day CoSynch + Controlled internal drug release (CIDR) + timed artificial insemination (TAI) in dairy heifers has resulted in adequate pregnancy rates compared with the 5‐day CoSynch + CIDR + TAI protocol. The objective of this study was to compare follicular growth, timing of ovulation and serum progesterone (P_4_), estradiol (E_2_) and luteinizing hormone (LH) concentrations in dairy heifers treated with modified 4‐ or 5‐day CoSynch + CIDR protocols (CIDR for 4 or 5 days, PGF_2_ *α* at CIDR removal and GnRH + TAI 72 h later). Twelve cycling Holstein heifers were randomly assigned to either the 4‐ or 5‐day Co‐Synch+CIDR (*n* = 6/treatment) to receive an intravaginal insert CIDR ^®^ containing 1.38 g of P_4_ for 4 or 5 days, respectively. At CIDR removal, 25 mg of PGF_2_ *α* was injected IM; 72 h after CIDR removal, heifers received 100 *μ*g of GnRH IM and timed artificial insemination (TAI). Follicular growth and timing of ovulation were assessed using transrectal ultrasonography. Blood samples were collected at the time of CIDR insertion and at frequent time points after CIDR removal for determination of P_4_ (at TAI), E_2_ (every 12 h) and LH (every 6 h during the first and second day and every 2 h on the third day). Heifers in the 4‐day group had smaller follicles from CIDR insert removal to ovulation compared with heifers in the 5‐day treatment. Five of six heifers (83.3%) in the 4‐day treatment ovulated at 90–96 h post CIDR insert removal, whereas most heifers in the 5‐day treatment (4/6; 66.6%) ovulated at 84–90 h post CIDR insert withdrawal. Heifers in the 5‐day treatment reached greater peak LH concentration between 48 and 72 h after CIDR insert removal and lesser E_2_ concentration at TAI than heifers in the 4‐day treatment. In conclusion, heifers in the 4‐day treatment had smaller follicular diameter at 0, 30, 36, 42 and 48 h after CIDR insert removal, longer interval from CIDR insert removal to ovulation, greater E_2_ concentrations at TAI, and lesser peak LH concentration than heifers in the 5‐day treatment. These results represent a baseline for further studies to determine if prolonging the interval to TAI by 6 h in the 4‐day CoSynch+CIDR would improve pregnancy risk.

## Introduction

Advances in ovulation synchronization represent a promising tool to improve reproductive performance and management of dairy heifers. Hormonal protocols using gonadotropin releasing hormone (GnRH) and prostaglandin F_2*α*_ (PGF_2*α*_) in combination or not with Controlled internal drug release (CIDR) insert for 7 days have been successfully applied in lactating dairy cows (El‐Zarkouny *et al*. 2004). However, poorer ovulation synchronization and pregnancy risk have been obtained in dairy heifers treated with these conventional synchronization protocols (Schmitt *et al*. 1996; Pursley *et al*. 1997; Rivera *et al*. 2004, 2005; Stevenson *et al*. 2008). This lesser response has been attributed to the faster follicular turnover, larger proportion of heifers with > 2 follicular waves, lesser ovulation risk, and greater percentage of heifers displaying premature oestrus compared with dairy cows (Sirois & Fortune 1988; Rivera *et al*. 2004; Sartori *et al*. 2004). A modified 5‐day CoSynch+CIDR protocol has been used in an attempt to improve pregnancy per timed AI (P/TAI) in dairy heifers. Treatment of dairy heifers with 5‐day CoSynch+CIDR protocol has resulted in adequate P/TAI ranging from 52% to 61% (Rabaglino *et al*. 2010).

Further studies found that the fertility of dairy heifers treated with the 5‐day CoSynch+CIDR protocol was not improved by an initial GnRH dose on Day 0 (Colazo & Ambrose 2011; Lima *et al*. 2011) or a second injection of PGF_2*α*_ 12 h after CIDR insert removal when GnRH is not administered at initiation of the protocol (Rabaglino *et al*. 2010).

A major factor limiting the development of these programmes is to overlook the importance of administering the hormonal injections at the prescribed day and time according to the specific protocol. A Monday to Friday 4‐day Cosynch+CIDR protocol has been investigated in dairy heifers in an attempt to simplify the routine reproductive management (Palomares *et al*. 2015). Heifers treated with the 4‐day protocol showed an adequate P/TAI (55.0%) which was not statistically different from that observed in the 5‐day CoSynch+CIDR (63.3%). Although this difference (55.0 vs. 63.3%) did not reach statistical significance, these results suggest that some differences in follicular development and timing of ovulation between treatments may be present. Serum E_2_ concentration at the time of AI (72 hours after CIDR insert removal) was greater (*P* < 0.01) in the 4‐day CoSynch+CIDR treatment than in the 5‐day CoSynch+CIDR treatment, suggesting differences in the stage of follicular development and steroidogenic capacity between treatments (Palomares *et al*. 2015).

Studies on follicular development and ovulation are warranted to determine the appropriate timing of AI that contribute to improve pregnancy risk in heifers treated with the 4‐day CoSynch+CIDR protocol. We hypothesized that heifers treated with 4‐day CoSynch+CIDR protocol have longer interval from CIDR insert removal to ovulation and different concentrations of estradiol (E_2_) and luteinizing hormone (LH) compared with heifers treated with a 5‐day CoSynch+CIDR protocol. Therefore, the objectives of this study were to compare follicular growth and timing of ovulation (interval from CIDR insert removal to ovulation) in dairy heifers treated with 4‐ or 5‐day CoSynch+CIDR, and determine their serum progesterone (P_4_), E_2_ and luteinizing hormone (LH) concentrations after CIDR insert removal.

## Materials and methods

### Heifers, diets and housing

The experimental protocols applied in this study were previously revised and approved by the Institutional Animal Care and Use Committee of the University of Georgia. The study was conducted at the University of Georgia, Dairy Teaching Farm, located in Athens, Georgia. The cycling heifers received a dose of 25 mg of PGF_2*α*_ (5 mL Lutalyse ^®^ Zoetis Animal Health) intramuscularly (IM) 11 days before initiation of the study. A total of 12 nulliparous cycling Holstein heifers, aged 13 to 15 months were enrolled. Heifers were managed in a barn facility with access to free stalls and pasture. The heifers were fed a total mixed ration twice daily that met or exceeded the nutritional requirements for Holstein heifers.

### Experimental design and treatments

**Figure 1:**
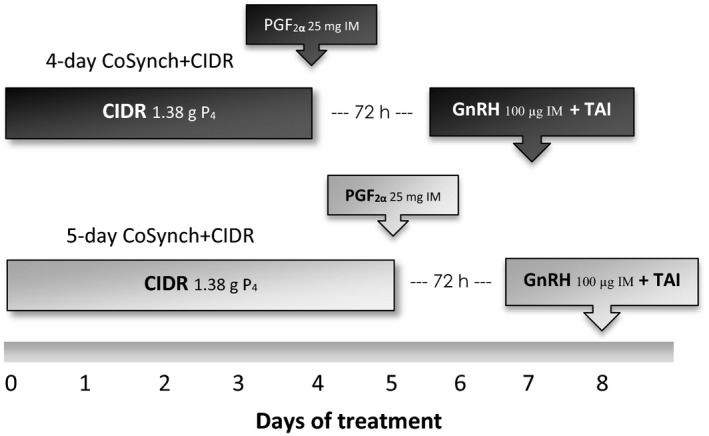
Diagrammatic representation of the 4‐ and 5‐day CoSynch + CIDR synchronization protocols. CIDR, controlled internal drug release; TAI, timed artificial insemination.

A total of 12 nulliparous cycling Holstein heifers were randomly assigned (six heifers per treatment) to one of two treatments:

1. 4‐day CoSynch+CIDR (*n* = 6): The heifers received an intravaginal CIDR insert (Eazi‐Breed CIDR^®^, Zoetis Animal Health, Florham Park, NJ, USA) containing 1.38 g of progesterone for 4 days. On the day of CIDR insert removal, 25 mg of PGF_2*α*_ (5 mL Lutalyse ^®^ Zoetis Animal Health) was injected IM; 72 h after CIDR insert removal, the heifers received 100 *μ*g of GnRH (2 mL Factrel^®^, Zoetis Animal Health) IM and TAI (Fig. 1).
2. 5‐day CoSynch+CIDR (*n* = 6): The heifers received an intravaginal CIDR insert containing 1.38 g of P_4_ for 5 days. The heifers were administered 25 mg of PGF_2α_ IM at the time of CIDR insert removal, and 100 *μ*g of GnRH IM and TAI 72 h after CIDR insert removal (Fig. 1).

### Ultrasonography

Transrectal ultrasonography was performed immediately before CIDR insertion using an ultrasound with a 5‐MHz probe (Ibex Pro E.I. Medical Imaging, CO, USA) to confirm the absence of reproductive pathologies. In addition, ovaries were scanned at the time of CIDR insertion, CIDR removal, and every 6 h after CIDR removal until ovulation. The location and diameter of follicles > 3.0 mm were mapped for tracking follicular growth. Time of ovulation was defined as the time in hours when the dominant follicle present at the time of GnRH treatment was no longer detected in the ovary. The interval from CIDR insert removal to the time of ovulation was calculated.

### Expression of oestrus

A pressure sensitive heatmount detector (Kamar; Kamar Proudcts, Inc., Zionsville, IN, USA) was glued onto the tailhead at the time of CIDR insert removal to assist in identifying heifers showing standing oestrus behaviour. Heifers with an activated (i.e. red colour) heatmount detector were considered to be in oestrus.

### Blood Sampling and plasma hormonal profile

Blood samples without anticoagulant were collected at the time of CIDR insertion, and TAI to determine serum P_4_ concentrations. Blood samples were also collected at the time of CIDR insert removal and every 12 h until the time of AI (72 h after CIDR removal) for analysis of E_2_ concentrations. In addition, blood samples were collected at 0, 12, 18, 24, 30, 36, 48, 50, 52, 54, 56, 58, 60, 66, 68, 70 and 72 h relative to the time of CIDR insert removal to determine the LH concentrations. A 14‐gauge × 14‐cm indwelling catheter (Abbocath‐T; Hospira Inc., Lake Forest, IL) was placed in the left jugular vein for the duration of intensive blood sampling. Before each sampling, approximately 5 mL of blood were collected and discarded for cleansing of the catheter. Samples were then collected using a 10‐mL syringe and transferred into vacutainer tubes for subsequent serum separation. After sampling, the catheters were flushed with heparinized solution (30 USP heparin sodium; Sigma‐Aldrich, Saint Louis, MO) to avoid coagulation. Samples were centrifuged at 2000*g* for 15 min at 4°C for serum separation and frozen at −20°C until shipping for analysis of P_4_, E_2_ and LH concentrations.

### Radioimmunoassay for progesterone (P_4_), 17β‐estradiol (E_2_) and Luteinizing Hormone (LH)

Progesterone, E_2_ and LH concentrations were measured using radioimmunoassay *(*RIA) at Colorado State University's Animal Reproduction and Biotechnology Laboratory. Progesterone and E_2_ were extracted separating the hormones from other constituents in the serum samples using petroleum ether and diethyl ether extraction/snap‐freeze techniques, respectively. Progesterone concentrations were measured in a single run using a RIA protocol developed at Colorado State University using iodinated progesterone ^125^I‐P4 (30 000–40 000 counts per minutes 100 *μ*L^−1^), rabbit progesterone antisera (1:48 000) and goat anti‐rabbit antibody (1:125). 17*β*‐Estradiol concentrations were measured in a single run using a commercial kit (ImmuChem Double Antibody 17*β*‐Estradiol ^125^I RIA kit, MP Biomedicals, Santa Ana, CA, USA). Luteinizing hormone concentrations were measured in a single run using a RIA protocol developed at Colorado State University utilizing iodinated Ovine LH ^125^I‐OLH (30 000–40 000 counts per minutes 100 *μ*L^−1^), rabbit LH antisera (1:40 000) and goat anti‐rabbit antibody (1:125).

Radioactivity was counted in a gamma spectrometer. The limits of detection were 0.025 ng mL^−1^, 0.55 pg mL^−1^ and 0.1 ng mL^−1^ for P_4_, E_2_ and LH, respectively. The intra‐assay coefficient of variation (CV) was 1.61, 7.82 and 4.61% for P_4_, E_2_ and LH, respectively.

### Statistical analysis

All statistical analyses were performed using a commercial statistical software (Statistical Analysis System, SAS 9.3; SAS Institute, Cary, NC, USA). Sample size was calculated using Proc Power on the basis of the expected means E_2_ concentration at TAI for each group (4.0 vs. 2.6 pg mL^−1^), the SDs (0.7 pg mL^−1^) of the means, with alpha = 0.05, and a statistical power of 90% (Palomares *et al*. 2015). A repeated measure analysis for a mixed generalized linear model was performed to compare the response variables follicular diameter and hormone (E_2_ and LH) concentrations overtime (baseline on hour 0 after CIDR removal and the Scheffé method to adjust for multiple comparisons) and between treatments. The onset (or end) of the LH peak was identified as the time point when the LH concentration had increased (or decreased) to a concentration ≥ (or ≤) the mean plus two standard deviations of the mean base line concentration in previous time points for a particular heifer (Nordéus *et al*. 2012). The time point of the LH peak was defined as the point with the highest LH concentration. For all analyses values of *P *≤* *0.05 were considered significant, and 0.05 < *P *≤* *0.1 was considered a tendency.

## Results

**Figure 2:**
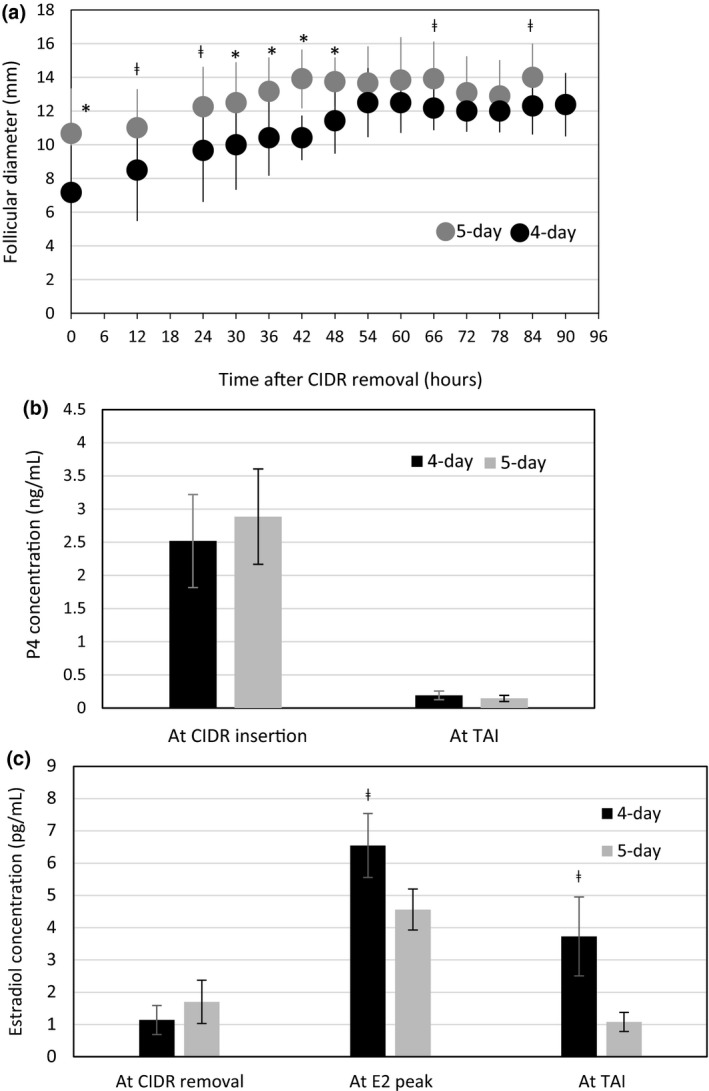
Diameter (mm) of the dominant follicle (a), serum mean concentration of progesterone (P_4_, ng mL^−1^; b) and estradiol (E_2,_ pg mL^−1^; c) in dairy heifers treated with 4‐ or 5‐ day CoSynch + CIDR synchronization protocols. For a: * Significant differences between groups at 0 (*P *=* *0.02), 30 (*P *=* *0.05), 36 (*P *=* *0.02), 42 (*P *=* *0.01) and 48 h (*P *=* *0.001) after CIDR removal. ^ǂ^Groups tended to be different (*P *=* *0.10). For b: Statistical difference was not observed between groups. For c: ^ǂ^ Mean E_2_ concentration tended to be different at E_2_ peak (*P *=* *0.1) and at TAI (72 h after CIDR removal, *P *=* *0.06) between treatments.

All heifers had a mature *corpus luteum* (CL) in the ovaries on the day of CIDR insertion. Furthermore, all animals showed follicular development (follicles of 3–10 mm of diameter) before initiating the treatments. No significant differences were found in the diameter of the follicles and CL between treatment groups, before starting the hormonal protocols. The diameter of the dominant follicle was significantly smaller in the heifers belonging to the 4‐day treatment at 0 (*P* = 0.02), 30 (*P* = 0.05), 36 (*P* = 0.02), 42 (*P* = 0.01) and 48 h (*P* = 0.001) after CIDR removal compared with heifers in the 5‐day treatment (Fig. 2a). Furthermore, dominant follicle diameter also tended (*P* ≤ 0.10) to be smaller in the 4‐day treatment at 12, 24, 66 and 84 h after CIDR insert removal and before ovulation (Fig. 2a). The follicular diameter at the time of CIDR removal, and before ovulation was 7.2 ± 2.8 and 12.3 ± 1.4 mm for the 4‐day treatment versus 10.7 ± 2.7 and 14.0 ± 2.0 mm for the 5‐day treatment.

Five of six heifers showed standing oestrus behaviour within 72 h after CIDR insert removal in the 5‐day treatment, while four of six animals did in the 4‐day treatment. All heifers showed a rise of E_2_ before ovulation (Table 1). The average time for a peak of E_2_ was 58 and 44 hours after CIDR insert removal and injection of PGF_2_ *α* in the 4‐ and 5‐day treatments, respectively (Table 2). In the 4‐day treatment, five of six heifers (83.3%) ovulated between 90 and 96 h after CIDR insert removal and one heifer did between 84 and 90 h post CIDR insert removal. In contrast, four of six heifers (66.6%) in the 5‐day treatment ovulated between 84 and 90 h post CIDR insert withdrawal; one heifer ovulated between 78 and 84 h after CIDR insert removal and one heifer did not ovulate during the experimental period (Table 1).

**Table 1:** Intervals (hours) from CIDR insert removal to E_2_ peak, LH peak and ovulation in dairy heifers treated with 4‐ or 5‐day CoSynch + CIDR protocols

Heifer #	Treatment Groups	Intervals from CIDR removal (h)		
		to E2 peak	To LH peak	To ovulation
1	4‐day	72	NA	90–96
2	4‐day	48	NA	90–96
3	4‐day	72	NA	90–96
4	4‐day	48	54	84–90
5	4‐day	60	60	90–96
6	4‐day	48	70	90–96
7	5‐day	48	66	84–90
8	5‐day	24	NA	NA
9	5‐day	60	66	84–90
10	5‐day	60	66	84–90
11	5‐day	24	NA	84–90
12	5‐day	48	50	78–84

NA: not available.

**Table 2:** Characteristics [duration (h), mean peak concentration (ng mL^−1^) and time of occurrence (h)] of LH and Estradiol in dairy heifers treated with 4‐ or 5‐ day CoSynch + CIDR synchronization protocols

End point	Treatment group		
	4‐day	5‐day	*P*‐value
Duration of the LH peak (h)	8.66 ± 1.76	13.5 ± 2.08	0.1
Mean LH peak concentration (ng mL^−1^)	13.3 ± 1.5	22.54 ± 2.7	0.04
Mean E_2_ peak concentration (pg mL^−1^)	6.54 ± 0.99	4.56 ± 0.63	0.1
Time to LH peak after CIDR removal (h)*	61.33 ± 4.6	63.6 ± 3.9	0.7
Time to E_2_ peak after CIDR removal (h)†	58.0 ± 4.8	44.0 ± 6.7	0.1

* Average time of the observed LH peak (*n* = 3 and 4 heifers in the 4‐day and 5‐day treatment groups, respectively).

† Average time of the observed E_2_ peak (*n* = 6 heifers in each treatment).

Almost all heifers in both treatments (11/12, 91.6%) showed high P_4_ concentration (P4 > 1 ng mL^−1^) on the day of initiation of treatments. Furthermore, serum P_4_ concentration at initiation of the protocols was not different between treatments (*P *>* *0.05; Fig. 2b). All heifers had low P_4_ concentration (<1 ng mL^−1^) at TAI. Serum E_2_ concentration increased after CIDR insert removal in both treatments; without statistical significance compared to baseline concentrations (data not shown). The 4‐day treatment tended to have greater E_2_ concentration during the E_2_ peak (*P *=* *0.1), and 72 h after CIDR insert removal (*P* = 0.06) compared with the 5‐day treatment (Fig. 2c).

**Figure 3:**
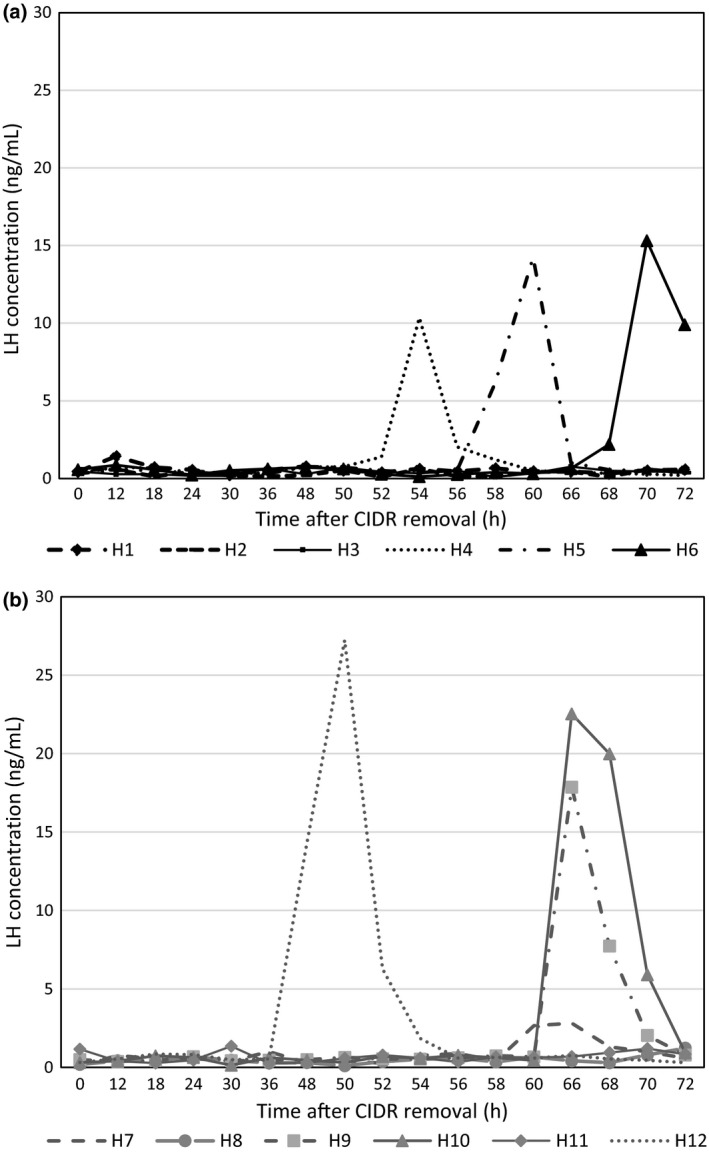
LH concentration (ng mL^−1^) after CIDR insert removal in dairy heifers treated with 4‐ (a) or 5‐ day CoSynch + CIDR (b) synchronization protocols. H: heifer

Heifers enrolled in the 5‐day treatment had more pronounced LH peaks than heifers in the 4‐day treatment. The mean peak LH concentration was greater in the heifers belonging to the 5‐day treatment than those in the 4‐day treatment (*P *<* *0.05). Moreover, the LH peak tended (*P* = 0.1) to stay longer above baseline values in the 5‐day treatment than the 4‐day treatment (Table 2). In the 5‐day treatment, four of six heifers (Heifers 7, 9, 10 and 12) had increased LH concentrations during the experimental period (Fig. 3b). An increase in LH concentration was detected in three of six heifers in the 4‐day treatment (Heifers 4, 5 and 6) between 54 and 72 h after CIDR insert removal compared to the baseline concentrations.

## Discussion

Heifers in the 4‐day treatment had smaller follicular diameter during the evaluation period and longer interval from CIDR insert removal to ovulation compared with heifers in the 5‐day treatment. Most of the heifers in the 4‐day treatment ovulated smaller follicles (12.3 mm) at 90 to 96 h after CIDR insert removal, compared with the high proportion of heifers in the 5‐day treatment, which ovulated larger follicles (14.0 mm) at 84 to 90 h post CIDR insert withdrawal. It is well documented that in cattle the timing of ovulation is influenced by the size of the preovulatory follicle (Sirois & Fortune 1988). Previous studies in dairy heifers submitted to ovulation synchronization protocols have shown a diameter of the ovulatory follicle between 13 and 14 mm (Sirois & Fortune 1988; Taponen *et al*. 1999; Nordéus *et al*. 2012; Ginther *et al*. 2013; Rantala & Taponen 2015), being similar to the heifers in the 5‐day treatment, but greater than the size observed in the 4‐day treatment. Other studies have reported larger follicular diameter in dairy heifers at CIDR insert removal (11.7 mm; Mellieon *et al*. 2012) and at ovulation (16.6 ± 0.9 mm Taponen *et al*. 1999) compared to the values reported here.

A positive correlation between the ovulatory follicle size and P_4_ secretion by the CL (Busch *et al*. 2008; Stevenson *et al*. 2008), ovulation and pregnancy risk has been observed in beef and dairy cattle (Vasconcelos *et al*. 1999; Perry *et al*. 2007; Sá Filho *et al*. 2010). In that regard, larger follicles contained more granulosa cells, resulting in a larger CL, with greater peripheral P_4_ concentrations (Oussaid *et al*. 2000; Perry *et al*. 2005). It is possible that the smaller ovulatory follicle size and longer interval to ovulation observed in the 4‐day treatment in the current study could explain the numerically lesser P/TAI previously reported in dairy (Palomares *et al*. 2015) and beef heifers (Fishman *et al*. 2015) treated with 4‐day CoSynch+CIDR compared with heifers submitted to the 5‐day protocol.

In 8 of 12 heifers the peak value of E_2_ concentration was reached between 48 and 60 h after injection of PGF_2_ *α* which was similar to previous reports (Taponen *et al*. 1999). A decrease in P_4_ and an increase in E_2_ are crucial in initiating an increase in LH release (Bleach *et al*. 2001; Perry 2012). This physiological event occurs concomitantly with a greater expression of gonadotropin receptors, steroidogenic enzymes and StAR in theca cells and/or granulosa cells (Bao & Garverick 1998), which results in the onset of oestrus and the activation of a positive feedback that causes the preovulatory FSH and LH surge (Bleach *et al*. 2001; Fortune *et al*. 2004). Later, the preovulatory LH surge alters dramatically the follicular steroidogenesis inhibiting expression of Inhibin A and aromatase Cyp19A1, leading to an abrupt decline in circulating Inhibin A and E_2_ concentrations (Bleach *et al*. 2001; Komar *et al*. 2001; Fortune *et al*. 2004). In the present study, a rise in E_2_ concentration preceded the LH peak and ovulation in most heifers of both treatment groups as previously reported (Haughian *et al*. 2004; Ginther *et al*. 2013).

Serum E_2_ concentrations tended to be greater at the time of AI and GnRH injection in the 4‐day treatment (despite having smaller follicle diameter) than the 5‐day treatment. Similarly, previous studies by our group using dairy (Palomares *et al*. 2015) and beef heifers (data not published) showed lesser E_2_ concentration at TAI in animals treated with 5‐ vs. 4‐day CoSynch+CIDR protocol. Strong correlations between size of the dominant follicle and intra‐follicular E_2_ concentrations during the preovulatory period have been reported previously (Ireland & Roche 1982; Kruip & Dieleman 1985). In the study by Jinks *et al*. (2013), even though there was a positive correlation between follicular size and E_2_ concentration (*r* = 0.45), follicle diameter was not always predictive of serum concentrations of E_2_, and 39% of cows with small follicles (<12.5 mm) had elevated E_2_ concentrations (≥8.4 pg mL^−1^) at the time of the second GnRH injection of the Ovsynch protocol. Follicular maturity is not precisely predicted by a single characteristic, but more probably predicted by the additive effect of many factors, such as length of proestrus, E_2_ production, diameter and age of the follicle and P_4_ production by the subsequent CL (Bridges *et al*. 2010). It is possible to speculate that in the present study the follicles with smaller diameter in the 4‐day treatment might have maintained their steroidogenic function for a longer period of time (before undergoing ovulation) than bigger follicles in the 5‐day treatment, resulting in numerically greater cumulative E_2_ concentrations measured in serum.

The mean peak LH concentration was greater in the heifers belonging to the 5‐day treatment than those in the 4‐day treatment. The current study had the limitations that blood samples were collected every 2 h from 48 until 72 h after CIDR insert removal instead every 30 or 60 min as done in previous studies to determine LH concentrations (Ginther *et al*. 2013). Moreover, the LH peaks occurred at variable time points of the experimental period in each heifer. These two factors limited the interpretation of temporal relationships between changes in LH and E_2_ concentrations and the time of ovulation (Ginther *et al*. 2013). In addition, blood samples were not collected at 62 and 64 h post CIDR insert removal, which prevented gathering complete information about the LH peak in heifer 7. A significant increase in LH concentration was not detected in three heifers in the 4‐day treatment (heifers 1, 2 and 3). These heifers ovulated between 90 and 96 h after CIDR insert withdrawal, and two of these animals had E_2_ peak at 72 h post insert removal (heifers 1 and 3). It is possible that these heifers had an LH surge > 72 h post CIDR insert removal. However, in the present study blood samples were not collected after this time point, limiting our ability to detect the preovulatory LH secretion after the administration of GnRH. Despite performing a power calculation analysis, we recognize that the number of animals per group was small, and therefore, care should be taken with the interpretation of these results.

In conclusion, heifers treated with the 4‐day CoSynch+CIDR protocol had smaller follicular diameter throughout the evaluation period, longer intervals from CIDR insert removal to ovulation (approximately 6 h longer), greater E_2_ concentration (during the E_2_ peak and at the time of AI). In addition, heifers in the 4‐day treatment appeared to have lesser LH peak concentration during the evaluation period than heifers in the 5‐day treatment. These results support our hypothesis that heifers treated with a 4‐day protocol require more time to complete follicular development and maturation after CIDR insert removal and PGF_2_ *α* injection, resulting in longer interval (6 h) from CIDR insert removal to ovulation compared with heifers treated with the 5‐day protocol. These results represent a baseline for further studies to determine if prolonging the interval to TAI by 6 h in the 4‐day CoSynch+CIDR protocol would improve pregnancy risk.

## Source of funding

Departments of Large Animal Medicine and Population Health, College of Veterinary Medicine, Department of Animal and Dairy Science, College of Agriculture, University of Georgia, Athens GA; and Zoetis Animal Health.

## Conflict of interest

The authors of this article have no conflicts of interest.

## Ethics statement

The authors confirm that the ethical policies of the journal, as noted on the journal's author guidelines page, have been adhered to and the appropriate ethical review committee approval has been received. The University of Georgia Institutional Animal Care and Use Committee (IACUC) guidelines were followed.

## Contributions

HFH and RAP contributed equally to this work during experimental design, sample collection, ultrasound evaluation, data interpretation and manuscript writing. AS, ER, MSF, DV, JHJB and JL contributed to ultrasound examination and blood sample collection and processing. MSF contributed to critical discussion and interpretation of the data. All the authors read and approved the manuscript.
